# Wheeled Chairs for Paralytics

**Published:** 1913-04-19

**Authors:** Godfrey H. Hamilton

**Affiliations:** Secretary. National Hospital, Queen Square, Bloomsbury, W.C., London


### Wheeled Chairs for Paralytics.

*To the Editor of* The Hospital.

Sir,—It is not often that the authorities of the London hospitals write to the press with any other object in view but to obtain the financial support of the public, and, while not implying that at the present time monetary assistance is not needed by us, I am asking for the hospitality of your columns in respect of another matter. Quite half of our patients are able to get up every day and to be taken to the recreation rooms, garden, chapel, or other parts of the building. As many of these patients have wholly or partially lost the use of their legs, a large number of wheeled chairs are required for their use. Many of our chairs have been mended over and over again, and are nearly worn out. As we have no spare cash to buy others, the present stock, already deficient, is likely to become more so.

I know it is often the case that there are wheeled chairs in private houses which are no longer needed for the purpose for which they were bought, and I venture to make an appeal to any of your readers who own such articles, and have no further use for them, to make a present of them to this hospital or its convalescent home adjoining East Finchley Station.—I am, yours faithfully,

Godfrey H. Hamilton, Secretary.

National Hospital,
Queen Square, Bloomsbury, W.C.,
London, April 11.

